# Enhanced Harmonic Partitioned Scheduling of Periodic Real-Time Tasks Based on Slack Analysis

**DOI:** 10.3390/s24175773

**Published:** 2024-09-05

**Authors:** Jiankang Ren, Jun Zhang, Xu Li, Wei Cao, Shengyu Li, Wenxin Chu, Chengzhang Song

**Affiliations:** 1 School of Computer Science and Technology, Dalian University of Technology, Dalian 116024, China; rjk@dlut.edu.cn (J.R.); lsyanling@mail.dlut.edu.cn (S.L.); 2 Key Laboratory of Social Computing and Cognitive Intelligence, Ministry of Education, Dalian 116024, China; 3 School of Computer Science and Technology, Xinjiang Normal University, Urumqi 830054, China; lixu@xjnu.edu.cn (X.L.); cwei@xjnu.edu.cn (W.C.); cwx12112024@163.com (W.C.); songchengzhang2024@163.com (C.S.); 4 Graduate School of Education, Dalian University of Technology, Dalian 116024, China

**Keywords:** Internet of Things, real-time scheduling, partitioned scheduling, multiprocessor systems, periodic real-time tasks, slack analysis

## Abstract

The adoption of multiprocessor platforms is growing commonplace in Internet of Things (IoT) applications to handle large volumes of sensor data while maintaining real-time performance at a reasonable cost and with low power consumption. Partitioned scheduling is a competitive approach to ensure the temporal constraints of real-time sensor data processing tasks on multiprocessor platforms. However, the problem of partitioning real-time sensor data processing tasks to individual processors is strongly NP-hard, making it crucial to develop efficient partitioning heuristics to achieve high real-time performance. This paper presents an enhanced harmonic partitioned multiprocessor scheduling method for periodic real-time sensor data processing tasks to improve system utilization over the state of the art. Specifically, we introduce a general harmonic index to effectively quantify the harmonicity of a periodic real-time task set. This index is derived by analyzing the variance between the worst-case slack time and the best-case slack time for the lowest-priority task in the task set. Leveraging this harmonic index, we propose two efficient partitioned scheduling methods to optimize the system utilization via strategically allocating the workload among processors by leveraging the task harmonic relationship. Experiments with randomly synthesized task sets demonstrate that our methods significantly surpass existing approaches in terms of schedulability.

## 1. Introduction

In Internet of Things (IoT) applications, sensors are critical components that continuously monitor and collect data from the environment. Generally, the sensor data need to be processed in real time to enable timely responses and actions [1,2,3,4]. With the rapid advancement in multiprocessor technology, multiprocessor platforms have already been widely adopted in IoT applications to handle large volumes of sensor data while maintaining real-time performance at a reasonable cost and with low power consumption [5,6,7,8]. Conversely, partitioned scheduling statically assigns tasks to specific processors, where they execute without migration.In comparison to global scheduling, partitioned scheduling can effectively alleviate the unpredictability of system run-time behaviors through safe isolation between different processors [9]. In most cases, the partitioning approach is generally considered to be the most appropriate solution owing to its simplicity and efficiency when the task set is predetermined and known in advance. Consequently, partitioned scheduling has gained widespread adoption and support in a variety of commercial real-time operating systems for IoT applications [10].

Unfortunately, the task allocation problem in partitioned scheduling is similar to the bin-packing problem, which is recognized as strongly NP-hard [11]. To address the task allocation problem in partitioned scheduling, a rich literature has been developed, and a comprehensive survey can be found in [12,13]. Early research in partitioned multiprocessor scheduling often relied on combining different bin-packing heuristics with task ordering methods for the task allocation [12,13]. However, these approaches disregard the potential impact of some implicit relations among tasks on the schedulability of tasks assigned to a processor [14]. More recently, there has been an increased focus on optimizing processor utilization by exploiting the task period relationship during task partitioning. Much work in this direction is motivated by the observation that the utilization bound of a task set tends to rise as the task periods exhibit greater harmonicity [15]. To optimize the schedulability of the system with periodic real-time tasks by leveraging the task period harmonic relationships, Wang et al. [14] proposed two harmonic-aware partitioned approaches to group tasks with harmonic periods on the same processor. For probabilistic real-time systems, Wang et al. [16] presented four harmonic metrics and a harmonicity-aware partitioning method to optimize system utilization. Their main idea was to assign harmonic tasks to a single processor to enhance the system usage by exploring the harmonic relationship among tasks. Nonetheless, its performance deteriorates when the system includes some heavy-utilization tasks. The main cause of this is the lack of consideration for workload characteristics during task allocation, resulting in a fragmentation issue, where certain tasks cannot be assigned despite the overall processor capacity being relatively high [17]. To address this problem, Ren et al. [17] proposed a workload-aware harmonic partitioned scheduling method for probabilistic multiprocessor real-time systems. For constrained-deadline real-time tasks, Wang et al. [18] introduced two harmonic scheduling methods, GIM and HCM. However, neither GIM nor HCM adequately incorporate the impact of harmonicity and workload characteristic on the task allocation performance. To address this problem, Ren et al. [19] further refined this approach by designing grouping metrics that consider both harmonicity and workload characteristics. These investigations demonstrate that system schedulability can be substantially enhanced compared to traditional bin-packing approaches when task harmonic relationships are effectively utilized. However, these studies quantify the task set harmonicity by measuring the “distance” from an associated harmonic task set with respect to change in density or utilization, and they construct a harmonic counterpart of a task set by scaling periods or deadlines. This harmonicity metric is too tight to effectively quantify the harmonicity of some task sets, limiting the potential improvements in schedulability for some task sets using existing harmonic-partitioned scheduling methods.

In this paper, we propose a more general harmonicity characterization approach based on the slack variation to address the limitations of the existing methods mentioned above. In particular, we propose an exact analysis method for the worst-case slack time and the best-case slack time to derive the harmonic index based on slack variation. With the proposed harmonic index, we present two enhanced harmonic task partitioning algorithms. The first algorithm, named EHAP-SV, focuses on improving the system utilization by leveraging the task harmonic relationships. To tackle the “fragmentation” issue and further enhance the system schedulability, we also propose a workload-aware harmonic partitioning algorithm called WAHP-SV, incorporating both harmonicity and workload characteristics into the scheduling framework.

**Contributions.** The primary contributions of this work are as follows:

- A more general harmonic index based on slack variation to effectively quantify the harmonicity degree of a periodic real-time task set;
- An exact analysis method for the worst-case slack time and the best-case slack time to derive the slack variation-based harmonic index;
- An enhanced harmonic task partitioning scheme to optimize the system utilization by leveraging the harmonic relationships to allocate harmonic tasks to one processor;
- A workload-aware harmonic task partitioning scheme by designing a metric integrating the exploration of harmonic relationships and workload characteristics to alleviate the “fragmentation” problem in the pure harmonic task partitioning method.

Experimental evaluation on randomly synthesized task systems demonstrates that our methods surpass existing partitioned scheduling approaches in terms of schedulability.

The remaining sections are structured as follows. Section 2 provides the system model and problem definition. In Section 3, we present the harmonic index based on slack variation and give its calculation method based on slack time analysis. Section 4 presents a detailed description of our proposed enhanced harmonic partitioned scheduling schemes. The experimental evaluation is given in Section 5, and we conclude the paper in Section 6.

## 2. System Model and Problem Definition

Before presenting the detailed system model, we summarize the major notations used in this paper in Table 1.

Sensors in IoT systems generally generate data at various rates to constantly track and measure some properties, and the processing tasks often have different periods and deadlines. In this paper, we focus on a real-time system in IoT with *n* independent periodic real-time sensor data processing tasks, represented by Γ = {$\tau_{1}$, …, $\tau_{n}$}. These tasks are preemptively scheduled on a multiprocessor platform comprising *m* identical processors with unit-capacity, indicated as Π= {$\pi_{1}$, …, $\pi_{m}$}, according to the rate monotonic (RM) policy. We assume a discrete notion of real time t=xθ, x≥0, where θ is both the unit time and the smallest unit of preemption, and all timing values are specified as non-negative integers. For each task $\tau_{i}\in \Gamma$, described by a triple $\tau_{i}=\left(C_{i},T_{i},D_{i}\right)$, where:

- $C_{i}$ represents the worst-case execution time (WCET) of $\tau_{i}$;
- $T_{i}$ denotes the inter-arrival time (period) of $\tau_{i}$;
- $D_{i}$ represents the relative deadline of $\tau_{i}$.

All tasks are considered to be implicit-deadline real-time tasks (i.e., $D_{i}=T_{i}$). All tasks are assumed to be initially released simultaneously at time t=0. The job of $\tau_{i}$ is indicated by $J_{i,k}$, with its release time represented by $r_{i,k}$. The WCETs of all tasks are assumed to be strictly positive. The worst-case response time (WCRT) of a task $\tau_{i}$, represented by $R_{i}$, measures the maximum interval between a job’s release and its completion, taking into account potential interference from other tasks. A task set Γ is considered schedulable if the WCRT of every task $\tau_{i}\in \Gamma$ does not exceed the deadline. The hyperperiod of the task set Γ is indicated by $H_{\Gamma }$, which is the least common multiple of periods of all tasks in Γ.

We characterize the workload of tasks by using the task utilization.

**Definition 1.**  *The* **utilization** *of a real-time task $\tau_{i}=\left(C_{i},T_{i},D_{i}\right)$, denoted as $U_{i}$, is expressed as*

(1) $$U_{i}=\frac{C_{i}}{T_{i}}.$$

**Definition 2.**  *The* **total utilization** *of a real-time task set* Γ*, indicated as $U_{\Gamma }$, is expressed as*

(2) $$U_{\Gamma }=\sum_{\tau_{i}\in \Gamma }U_{i}.$$

**Definition 3.**  *Given a set of n independent periodic real-time sets* Γ *= {$\tau_{1}$, …, $\tau_{n}$} and m identical processors Π= {$\pi_{1}$, …, $\pi_{m}$}, the goal of the* **partitioned multiprocessor scheduling problem** *is to assign each task in* Γ *to exactly one processor in* Π *such that all tasks meet their deadlines while minimizing the overall number of processors utilized.*

Note that the task allocation problem in partitioned scheduling is similar to the bin-packing problem, which is recognized as strongly NP-hard [11].

**Definition 4**  ([11]). *Given a bin capacity V and a set of n items $x_{1}$, …, $x_{n}$ with sizes $a_{1}$, …, $a_{n}$, the goal of the* **bin-packing optimization problem** *is to assign each item to a bin such that the number of bins is minimized and no bin exceeds its capacity V.*

**Theorem 1.**  *Given a set of n independent periodic real-time sets* Γ *= {$\tau_{1}$, …, $\tau_{n}$} and m identical processors Π= {$\pi_{1}$, …, $\pi_{m}$}, the partitioned multiprocessor scheduling problem is NP-hard.*

**Proof.**  To prove that the partitioned multiprocessor scheduling problem is NP-hard, we reduce an instance of the bin-packing problem to an instance of the partitioned multiprocessor scheduling problem.

For each item $x_{i}$ in the bin-packing problem, we create a corresponding task $\tau_{i}$ with WCET $C_{i}=a_{i}$. Let us consider the period and deadline of each task $\tau_{i}$ to be bin capacity *V* (i.e., $T_{i}=D_{i}=V$). This implies that each task must complete its execution within one period, which corresponds to the capacity of the bin. Each processor in the scheduling problem is considered as a bin in the bin-packing problem. Each processor has a capacity in terms of the total execution time it can handle within the period. Thus, if we can find a valid partitioned multiprocessor schedule for the tasks such that all tasks meet their deadlines, then this corresponds to a valid bin-packing solution, where the number of processors used is equivalent to the number of bins used.

According to [11], the bin-packing problem is NP-hard. Since we can reduce any instance of the bin-packing problem to an instance of the partitioned multiprocessor scheduling problem in polynomial time, and a solution to the scheduling problem would provide a solution to the bin-packing problem, it follows that the partitioned multiprocessor scheduling problem is also NP-hard.    □

To improve the performance of the partitioned scheduling via the harmonic relationship exploration based on the effective harmonicity quantification, we introduce a harmonic index based on slack variation to measure the harmonic characteristic among tasks. In the following, we provide some definitions of slack time.

**Definition 5.**  *For a job $J_{i,k}$ of a real-time task $\tau_{i}$, its* **slack time** *, indicated as $S_{i,k}$, is defined as the amount of idle time during the interval $\left[r_{i,k},r_{i,k}+T_{i}\right)$ when all jobs of all tasks always experience worst-case execution times.*

**Definition 6.**  *The* **worst-case slack time** *of a real-time task $\tau_{i}$, indicated as $S_{i}^{W}$, is defined as*

(3) $$S_{i}^{W}=\underset{k}{\inf}S_{i,k}.$$

**Definition 7.**  *The* **best-case slack time** *of a real-time task $\tau_{i}$, indicated as $S_{i}^{B}$, is expressed as*

(4) $$S_{i}^{B}=\underset{k}{\sup}S_{i,k}.$$

The primary aim of this research is to develop an efficient task partitioning method, ensuring that each processor’s assigned set of tasks is schedulable while minimizing the overall number of processors utilized. We analyze the drawbacks of the current harmonic index for harmonicity-aware partitioning schemes with an example, and introduce a novel harmonic index based on slack variation to guide the task partition. With the proposed harmonic index, we devise two efficient partitioned scheduling schemes to enhance system utilization through efficiently leveraging the task harmonic relationship.

## 3. Harmonicity Quantification Based on Slack Analysis

To enhance the performance of the partitioned scheduling via the harmonic relationship exploration based on the effective harmonicity quantification, we propose a slack variation-based harmonic index to guide the task partition. In this section, we first illustrate the motivation behind the slack variation-based harmonic index through an example to highlight the drawbacks of the utilization change-based harmonic index. We then provide a comprehensive explanation of our harmonic index based on slack variation. Additionally, we also present an exact analysis method to determine the worst-case and best-case slack times, which are utilized to calculate the harmonic index based on slack variation.

### 3.1. Motivations

In order to optimize task partition by exploring harmonic relationships, it is crucial to quantify the task set harmonicity degree, as not all tasks are strictly harmonic. Fan et  al. [14] presented a task set harmonic index through measuring the “distance” between a task set and the corresponding harmonic task set with regard to the total utilization change. However, such a harmonic index is too tight to effectively quantify the degree of harmonic for certain task sets, thereby limiting the potential improvement in the schedulability through the harmonic relationship exploration with this index. Here, we first discuss the shortcomings of the existing harmonic index, and then, introduce our proposed harmonic index based on slack variation.

**Definition 8**  ([14]). *For a task set $\Gamma =\{\tau_{1},\ldots ,\tau_{n}\}$, where $\tau_{i}=\left(C_{i},T_{i},D_{i}\right)$, we define a new task set $\Gamma^{\prime }=\left\{\tau_{1}^{\prime },\ldots ,\tau_{n}^{\prime }\right\}$, where $\tau_{i}^{\prime }=\left(C_{i},T_{i}^{\prime },D_{i}^{\prime }\right)$, $T_{i}^{\prime }\leq T_{i}$, $D_{i}^{\prime }=T_{i}^{\prime }$, and for any two tasks $\tau_{i}^{\prime }$ and $\tau_{j}^{\prime }$, if $T_{i}^{\prime }<T_{j}^{\prime }$, then $T_{j}^{\prime }$ is an integer multiple of $T_{i}^{\prime }$. This new task set $\Gamma^{\prime }$ is referred to as a* **sub-harmonic task set** *of task set* Γ*.*

**Definition 9**  ([14]). *Given a sub-harmonic task set $\Gamma^{\prime }$ of task set* Γ*, we define task set $\Gamma^{\prime }$ as* **the primary harmonic task set** *of task set* Γ *when there is no other sub-harmonic task set $\Gamma^{\prime \prime }$, such that $T_{i}^{\prime }\leq T_{i}^{\prime \prime }$ for all i where 1≤i≤n.*

According to [15], we can derive Theorem 2.

**Theorem 2**  ([15]). *Given a primary harmonic task set $\Gamma^{\prime }$ of task set* Γ*,* Γ *is schedulable on a uniprocessor under the RM policy when $U_{\Gamma^{\prime }}\leq 1$.*

**Definition 10**  ([14]). *For a task set* Γ*, let G(Γ) be all primary harmonic task sets of task set* Γ*. The* **utilization change-based harmonic index** *of task set* Γ*, indicated as $H^{\mathrm{uc}}\left(\Gamma \right)$, is expressed as*

(5) $$H^{\mathrm{uc}}\left(\Gamma \right)=\min_{\Gamma^{\prime }\in G\left(\Gamma \right)}U_{\Gamma^{\prime }}-U_{\Gamma }.$$

From Equation (5), we can observe that $H^{\mathrm{uc}}$ is characterized as the minimum “distance” between a task set and its primary harmonic task sets with regard to the change in total utilization. We can also see that a low value of $H^{\mathrm{uc}}$ indicates a high harmonic relationship, and a task set Γ is strictly “harmonic” exactly when $H^{\mathrm{uc}}\left(\Gamma \right)=0$. For primary harmonic task sets, they can be obtained with the *Sr* or *DCT* algorithms in [15]. However, the original intention of the primary harmonic task set is to improve the schedulability analysis of the RM scheduling strategy, and it considers a task set to be strictly harmonic exactly when every pair of tasks in the task set are strictly harmonic. This leads to inaccurate quantification of the harmonicity of some task sets from the perspective of system resource utilization, and we illustrate this issue with an example.

**Figure 1:**
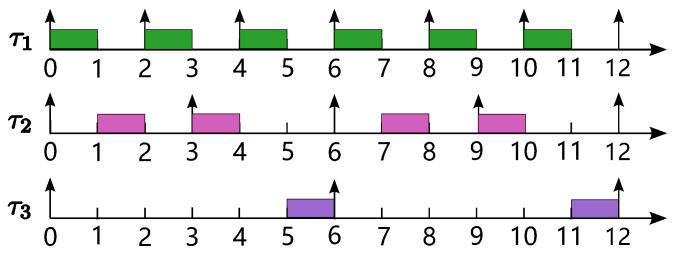
Simulation of an SAS for $\left\{\tau_{1},\tau_{2},\tau_{3}\right\}$ given in Table 2.

**Example 1.**  *Let us consider a task set $\Gamma =\{\tau_{1},\tau_{2},\tau_{3}\}$, as given in Table 2, executed on a uniprocessor under the RM scheduling policy. Here, $T_{i}^{\prime }$ represents the period of the task corresponding to task $\tau_{i}$ in a primary harmonic task set $\Gamma^{\prime }$ of* Γ*, ensuring that $U_{\Gamma^{\prime }}-U_{\Gamma }$ is minimized. We can find that $U_{\Gamma }=1/2+1/3+1/6=1$ and $U_{\Gamma^{\prime }}=2/3+1/3+1/6=7/6$, and thus, $H^{\mathrm{uc}}\left(\Gamma \right)=7/6-1=1/6$. Therefore,* Γ *is not strictly "harmonic" based on the definition of the harmonic index $H^{\mathrm{uc}}$. However, according to the simulation of the synchronous arrival sequence (SAS) for all tasks in* Γ *in Figure 1, it is evident that* Γ *is schedulable. Moreover, the utilization of* Γ *is 1, and thus,* Γ *can be regarded as strictly “harmonic” from the perspective of system resource utilization because the maximum utilization is achieved. Therefore, the harmonic index $H^{\mathrm{uc}}$ is not able to effectively quantify the harmonicity degree of* Γ *due to its tight restriction. This leads to the harmonic partitioned scheduling method based on $H^{\mathrm{uc}}$ not offering good performance for some task sets (see Example 3). In the following section, we give a more general harmonic index based on slack variation, which can effectively quantify the harmonicity of task set* Γ *(see Example 2).*

### 3.2. Harmonic Index Based on Slack Variation

As indicated in Example 1, the utilization change-based harmonic index cannot effectively quantify the harmonicity of some task sets from the perspective of system resource utilization. Now, we first discuss the property of the schedulable task set that can achieve maximum utilization; thus, we can shed some light on the design of our proposed harmonic index based on slack variation.

**Lemma 1.**  *Consider a schedulable task set $\Gamma =\{\tau_{1},\ldots ,\tau_{n}\}$ with $U_{\Gamma }=1$ on a uniprocessor under the rate monotonic (RM) policy. Here, tasks in* Γ *are sorted in decreasing priority and their WCETs are greater than 0. Then, the WCRT $R_{n}$ of $\tau_{n}$ equals its period $T_{n}$, that is, $R_{n}=T_{n}$.*

**Proof.**  Since tasks in task set Γ are ordered in decreasing priority, task $\tau_{n}$ has the lowest priority and $T_{1}\leq T_{2}\leq \ldots \leq T_{n}$. Since $U_{\Gamma }=1$, we can obtain

(6) $$\frac{C_{1}}{T_{1}}+\frac{C_{2}}{T_{2}}+\ldots +\frac{C_{n}}{T_{n}}=1.$$

Thus, we can obtain

(7) $$\sum_{1\leq i\leq n-1}\frac{T_{n}*C_{i}}{T_{i}}+C_{n}=T_{n}.$$

Based on [20], the WCRT $R_{n}$ satisfies the following equation:

(8) $$R_{n}=\sum_{1\leq i\leq n-1}\left\lceil \frac{R_{n}}{T_{i}}\right\rceil *C_{i}+C_{n}.$$

Because the task set Γ is schedulable, we have $R_{n}=T_{n}-\delta$, where δ≥0. Therefore, with Equations (7) and (8), we have

(9) $$\begin{array}{cc} T_{n}-\delta & =\sum_{1\leq i\leq n-1}\left\lceil \frac{T_{n}-\delta }{T_{i}}\right\rceil *C_{i}+C_{n}\\ & \geq \sum_{1\leq i\leq n-1}\frac{T_{n}-\delta }{T_{i}}*C_{i}+C_{n}\\ \; & =T_{n}-\delta *\sum_{1\leq i\leq n-1}\frac{C_{i}}{T_{i}}. \end{array}$$

By Equation (9), we have

(10) $$\delta *\left(1-\sum_{1\leq i\leq n-1}\frac{C_{i}}{T_{i}}\right)\leq 0.$$

With Equations (6) and (10), we have

(11) $$\delta *\frac{C_{n}}{T_{n}}\leq 0.$$

Since the WCETs of all tasks are greater than 0 and δ≥0, by Equation (11), we have δ=0. Therefore, the worst-case response time $R_{n}$ is equal to its period $T_{n}$.    □

Based on Lemma 1, for a schedulable task set $\Gamma =\{\tau_{1},\ldots ,\tau_{n}\}$ with $U_{\Gamma }=1$ on a uniprocessor under the RM policy, we can obtain the relationship between the period of task $\tau_{n}$ and the periods of tasks with higher priorities than task $\tau_{n}$ (i.e., Lemma 2).

**Lemma 2.**  *Consider a schedulable task set $\Gamma =\{\tau_{1},\ldots ,\tau_{n}\}$ with $U_{\Gamma }=1$ on a uniprocessor under the rate monotonic (RM) policy. Here, tasks in* Γ *are sorted in descending priority and their WCETs are greater than 0. Then, the period $T_{n}$ of task $\tau_{n}$ is a common multiple of periods of all higher-priority tasks $\tau_{i}$ for 1≤i≤n−1.*

**Proof.**  By Lemma 1 and Equation (8), we have

(12) $$T_{n}=\sum_{1\leq i\leq n-1}\left\lceil \frac{T_{n}}{T_{i}}\right\rceil *C_{i}+C_{n}.$$

By Equations (7) and (12), we have

(13) $$\sum_{1\leq i\leq n-1}\left(\left\lceil \frac{T_{n}}{T_{i}}\right\rceil -\frac{T_{n}}{T_{i}}\right)*C_{i}=0.$$

Since the WCETs of all tasks are greater than 0, by Equation (13), we have $\left\lceil T_{n}/T_{i}\right\rceil =T_{n}/T_{i}$ for each task $\tau_{i}$. Therefore, the period $T_{n}$ of task $\tau_{n}$ is a common multiple of periods of all higher-priority tasks $\tau_{i}$ for 1≤i≤n−1.   □

**Theorem 3.**  *Consider a schedulable task set $\Gamma =\{\tau_{1},\ldots ,\tau_{n}\}$ with $U_{\Gamma }=1$ on a uniprocessor under the rate monotonic (RM) policy. Here, tasks in* Γ *are sorted in decreasing priority and their WCETs are greater than 0. The worst-case slack time $S_{n}^{W}$ of $\tau_{n}$ equals its best-case slack time $S_{n}^{B}$, i.e., $S_{n}^{W}=S_{n}^{S}$.*

**Proof.**  According to Lemma 2, the period $T_{n}$ of task $\tau_{n}$ is a common multiple of periods of all higher-priority tasks $\tau_{i}$ (1≤i≤n−1). Moreover, because task $\tau_{n}$ is the lowest-priority task in Γ, its period $T_{n}$ equals the hyperperiod of Γ (i.e., $T_{n}=H_{\Gamma }$). Since all tasks in Γ are initially released at the same time, each job $J_{n,k}$ of task $\tau_{n}$ will be released concurrently with a job of every higher-priority task $\tau_{i}$ (1≤i≤n−1). Therefore, all jobs of task $\tau_{n}$ have the same slack times, and thus, the worst-case slack time $S_{n}^{W}$ of $\tau_{n}$ equals its best-case slack time $S_{n}^{B}$.   □

From Theorem 3, we can find that for a schedulable task set under RM scheduling policy that achieves maximum utilization (i.e., 1), all jobs of the lowest-priority task share the same slack times (i.e., the worst-case slack time is equal to the best-case slack time). On the basis of this observation, we introduce the harmonic index based on the slack variation as follows.

**Definition 11.**  *Consider a schedulable task set $\Gamma =\{\tau_{1},\ldots ,\tau_{n}\}$ on a uniprocessor under the rate monotonic (RM) policy. Here, tasks in* Γ *are sorted in decreasing priority. The* **slack variation-based harmonic index** *of the task set* Γ*, indicated as $H^{\mathrm{slack}}\left(\Gamma \right)$, is expressed as*

(14) $$H^{\mathrm{slack}}\left(\Gamma \right)=\frac{S_{n}^{B}-S_{n}^{W}}{T_{n}}.$$

*where $S_{n}^{B}$ and $S_{n}^{W}$ are the best-case and worst-case slack times of $\tau_{n}$, respectively.*

A low value of $H^{\mathrm{slack}}$ implies a high harmonicity degree, and the task set Γ is “harmonic” if $H^{\mathrm{slack}}\left(\Gamma \right)=0$. When $H^{\mathrm{slack}}\left(\Gamma \right)>0$, the index $H^{\mathrm{slack}}\left(\Gamma \right)$ can be viewed as a measure of processor resource waste when the lowest-priority task achieves the largest schedulable WCET, since the worst-case situation (i.e., the case where a job of the task experiences its WCET) should be considered for the schedulability analysis. Therefore, a higher utilization can be attained by assigning tasks with a lower slack variation-based harmonic index to the same processor to balance the slack time.

**Example 2.**  *Let us consider task set* Γ *as depicted in Table 2 again. From Figure 1, we can find that the* Γ *is schedulable on a uniprocessor, and $S_{3}^{W}=S_{3}^{B}=1$ for the lowest-priority task $\tau_{3}$. Thus, we can obtain $H^{\mathrm{slack}}\left(\Gamma \right)=0$. Therefore,* Γ *is strictly harmonic according to the slack variation-based harmonic index. Consider the sub-task set $\Gamma^{\mathrm{sub}}=\left\{\tau_{1},\tau_{2}\right\}\subset \Gamma$. For the lowest-priority task $\tau_{2}$ in $\Gamma^{\mathrm{sub}}$, $S_{2}^{W}=1$ and $S_{2}^{B}=2$, and thus, $H^{\mathrm{slack}}\left(\Gamma^{\mathrm{sub}}\right)=\left(2-1\right)/3=1/3>0$. Therefore, the task set $\Gamma^{\mathrm{sub}}$ is not harmonic according to the harmonic index based on slack variation. Note that for the task set $\Gamma^{\mathrm{sub}}$, task $\tau_{2}$ achieves the largest schedulable WCET even though there is still some slack for some jobs (e.g., jobs $J_{2,2}$ and $J_{2,4}$), since the worst-case scenarios (i.e., jobs $J_{2,1}$ and $J_{2,3}$) should be considered for the schedulability analysis.*

**Corollary 1.**  *Consider a schedulable task set $\Gamma =\{\tau_{1},\ldots ,\tau_{n}\}$ on a uniprocessor under the rate monotonic (RM) policy, where the WCETs of all tasks are greater than 0. If the utilization change-based harmonic index $H^{\mathrm{uc}}\left(\Gamma \right)=0$, then the slack variation-based harmonic index $H^{\mathrm{slack}}\left(\Gamma \right)=0$.*

**Proof.**  Suppose that tasks in Γ are sorted in decreasing priority under the RM policy, and thus, $T_{1}\leq T_{2}\leq \ldots \leq T_{n}$. Since $H^{\mathrm{uc}}\left(\Gamma \right)=0$, $T_{j}$ is an exact multiple of $T_{i}$ for any two tasks $\tau_{i}$ and $\tau_{j}$ in the task set Γ if $T_{i}\leq T_{j}$. Hence, $T_{n}$ is divisible by $T_{i}$ for any task $\tau_{i}\in \Gamma$, and thus, $T_{n}$ is a common multiple of periods of all higher-priority tasks $\tau_{i}$ for 1≤i≤n−1. Therefore, similar to the proof for Theorem 3, we can obtain that the worst-case slack time $S_{n}^{W}$ of task $\tau_{n}$ equals its best-case slack time $S_{n}^{B}$. Thus, we can obtain $H^{\mathrm{slack}}\left(\Gamma \right)=0$.    □

Note that a strictly harmonic task set (e.g., task set Γ depicted in Table 2) according to harmonic index $H^{\mathrm{slack}}$ may not be strictly harmonic according to the harmonic index $H^{\mathrm{uc}}$ (see Examples 1 and 2). Thus, from Corollary 1, we can find that a strictly harmonic task set according to the harmonic index $H^{\mathrm{uc}}$ is just a special case of a strictly harmonic task set according to the harmonic index $H^{\mathrm{slack}}$. Therefore, the harmonic index $H^{\mathrm{slack}}$ is more general than the harmonic index $H^{\mathrm{uc}}$, and it can more effectively quantify the harmonicity.

### 3.3. Slack Time Analysis

In order to calculate the slack variation-based harmonic index $H^{\mathrm{slack}}$ for a task set Γ, we should analyze the worst-case and best-case slack times for the lowest-priority task in Γ. Now, we first identify the job of a task that experiences the worst-case slack time with Theorem 4.

**Theorem 4.**  *A job of a task experiences the worst-case slack time when it is released simultaneously with the jobs of all higher-priority tasks.*

**Figure 2:**
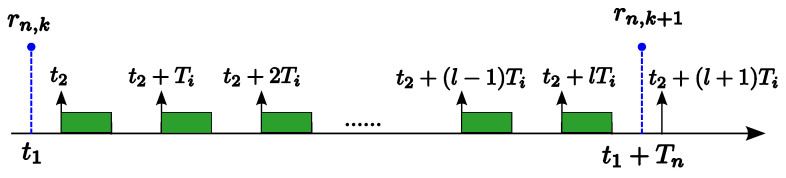
Scenario illustration for the proof of Theorem 4.

**Proof.**  Consider a task set $\left\{\tau_{1},\ldots ,\tau_{n}\right\}$ ordered in decreasing priority under the RM policy. Let $J_{n,k}$ be a job of task $\tau_{n}$ that is released at $t_{1}$ (i.e., $r_{n,k}=t_{1}$). As illustrated in Figure 2, we consider that the jobs of a higher-priority task $\tau_{i}$ (1≤i≤n−1) are released at $t_{2},t_{2}+T_{i},t_{2}+2T_{i},\ldots ,t_{2}+lT_{i}$ during the interval $\left[t_{1},t_{1}+T_{n}\right)$. From Figure 2, we can find that shifting the instance release time $t_{2}$ of task $\tau_{i}$ forward does not increase the slack time for job $J_{n,k}$ until $t_{2}=t_{1}$. Therefore, the slack time for job $J_{n,k}$ will be minimized when $t_{2}$ coincides with $t_{1}$. The theorem can be proved by repeating this argument for all higher-priority tasks $\tau_{i}$ (1≤i≤n−1).    □

Similarly, we can identify the job of a task that experiences the best-case slack time with the following theorem.

**Theorem 5.**  *A job of a task experiences the best-case slack time whenever its succeeding job is released concurrently with the jobs of all tasks with higher priorities.*

**Figure 3:**
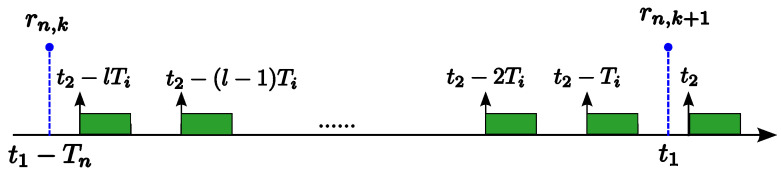
Scenario illustration for the proof of Theorem 5.

**Proof.**  Let $\left\{\tau_{1},\ldots ,\tau_{n}\right\}$ be a task set ordered in decreasing priority under the RM policy. Consider a job $J_{n,k}$ of $\tau_{n}$ that is released at $t_{1}-T_{n}$ and its succeeding job $J_{n,k+1}$ is released at $t_{1}$ (i.e., $r_{n,k}=t_{1}-T_{n}$ and $r_{n,k+1}=t_{1}$). As illustrated in Figure 3, we consider that the earliest job of a higher-priority task $\tau_{i}$ (1≤i≤n−1) released not before $t_{1}$ is released at $t_{2}$ (i.e., $t_{1}\leq t_{2}$). From Figure 3, we can find that shifting the instance release time $t_{2}$ of task $\tau_{i}$ forward does not reduce the slack time for job $J_{n,k}$ until $t_{2}=t_{1}$. Therefore, the slack time for job $J_{n,k}$ will be maximized when $t_{2}$ coincides with $t_{1}$. The theorem can be proved by repeating this argument successively for all higher-priority tasks $\tau_{i}$ (1≤i≤n−1).    □

Before giving the calculation method for the worst-case slack time and the best-case slack time, we first introduce some definitions about the level-*i* active period given in [21].

For a task $\tau_{i}$, its *pending load* $PL_{i}\left(t\right)$ refers to the processing workload that must be finished by time *t* for the jobs with priorities equal to or higher than that of task $\tau_{i}$ that are activated before *t*. A level-*i* active period is defined as the time interval $\left[t_{s},t_{e}\right)$ which meets the conditions $PL_{i}\left(t_{s}\right)=0$, $PL_{i}\left(t_{e}\right)=0$, and $PL_{i}\left(t\right)>0$ for every time $t\in (t_{s},t_{e})$. According to [21], for fixed-priority real-time systems, the worst-case duration $WL_{i}$ of a level-*i* active period is the minimum positive real number $x\in R^{+}$ that satisfies the condition

(15) $$x=\sum_{1\leq j\leq i}\left\lceil \frac{x}{T_{j}}\right\rceil *C_{j}.$$

Note that we can obtain $WL_{i}$ through an iterative process for Equation (15), beginning with a lower bound.

**Theorem 6.**  *Consider a schedulable task set $\Gamma =\{\tau_{1},\ldots ,\tau_{n}\}$ on a uniprocessor under the rate monotonic (RM) policy. Here, tasks in* Γ *are ordered in decreasing priority. Let $J_{n}^{W}$ be a job of task $\tau_{n}$ released concurrently with the jobs of all tasks with higher priorities, and let $r_{n}^{W}$ denote its release time. Let $r_{n}^{W}+t_{\mathrm{end}}$ be the end time of the available slack for job $J_{n}^{W}$ during the interval $\left[r_{n}^{W},r_{n}^{W}+T_{n}\right)$. Then, the amount of available slack time for $J_{n}^{W}$ can be formulated as*

(16) $$S_{n}^{W}=t_{\mathrm{end}}-\sum_{i<n}\left\lceil \frac{t_{\mathrm{end}}}{T_{i}}\right\rceil *C_{i}.$$

**Figure 4:**
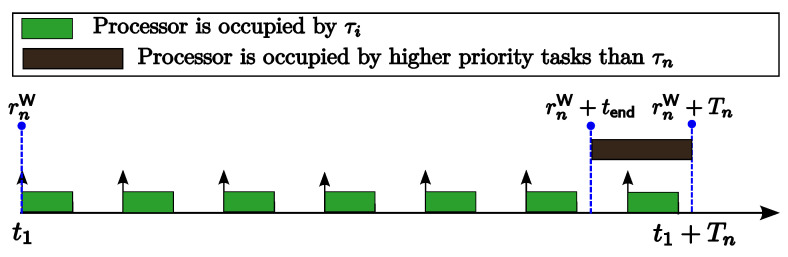
Workload of higher-priority tasks $\tau_{i}$ (i<n) during $\left[r_{n}^{W},r_{n}^{W}+T_{n}\right)$.

**Proof.**  Suppose that $J_{n}^{W}$ is released concurrently with the jobs of all tasks with higher priorities at time $t_{1}$. According to Theorem 4, job $J_{n}^{W}$ will experience the worst-case slack time. Figure 4 shows the workload of higher-priority tasks $\tau_{i}$ (i<n) during $\left[r_{n}^{W},r_{n}^{W}+T_{n}\right)$. Since $r_{n}^{W}+t_{\mathrm{end}}$ is the end time of the available slack for job $J_{n}^{W}$ during the interval $\left[r_{n}^{W},r_{n}^{W}+T_{n}\right)$, from Figure 4, we can observe that all the higher-priority jobs released no later than $r_{n}^{W}+t_{\mathrm{end}}$ are completed before $r_{n}^{W}+t_{\mathrm{end}}^{W}$, and there is no available slack for $J_{n}^{W}$ during the interval $\left[r_{n}^{W}+t_{\mathrm{end}},r_{n}^{W}+T_{n}\right)$. Furthermore, because job $J_{n}^{W}$ is released concurrently with the jobs of all tasks with higher priorities, for each highest-priority task $\tau_{i}$ (i<n), the count of jobs that need to be executed during the interval $\left[r_{n}^{W},r_{n}^{W}+t_{\mathrm{end}}^{W}\right)$ is $\left\lceil t_{\mathrm{end}}/T_{i}\right\rceil$. Thus, the workload of tasks with higher priorities during the interval $\left[r_{n}^{W},r_{n}^{W}+t_{\mathrm{end}}^{W}\right)$ is $\sum_{i<n}\left\lceil t_{\mathrm{end}}/T_{i}\right\rceil *C_{i}$. Therefore, the amount of available slack for job $J_{n}^{W}$ can be obtained with Equation (16).   □

According to Theorem 6, we can obtain the worst-case slack time of $\tau_{n}$ through searching time $t_{\mathrm{end}}$ for job $J_{n}^{W}$. Since all tasks are released at the same time t=0 in the beginning, we can consider $r_{n}^{W}=0$ for job $J_{n}^{W}$. Given the worst-case length $WL_{n-1}$ of a level-n−1 active period, there must be some slack for job $J_{n}^{W}$ during the interval $\left[T_{n}-WL_{n-1},T_{n}\right)$. Therefore, $t_{\mathrm{end}}$ for job $J_{n}^{W}$ can be expressed as follows:

(17) $$t_{\mathrm{end}}=\underset{t\in [T_{n}-WL_{n-1},T_{n}]}{\arg\max}t-\sum_{i<n}\left\lceil \frac{t}{T_{i}}\right\rceil *C_{i}.$$

Since $t_{\mathrm{end}}$ is the end time of the last available slack for job $J_{n}^{W}$ with $r_{n}^{W}=0$ during the interval $\left[0,T_{n}\right)$, a higher-priority task must be invoked at time $t_{\mathrm{end}}$. Therefore, the search space for $t_{\mathrm{end}}$ can be reduced to the invoking points of higher-priority tasks during the interval $\left[T_{n}-WL_{n-1},T_{n}\right)$. Let $V_{n}^{W}$ denote the search space for $t_{\mathrm{end}}$; it can be expressed as follows:

(18) $$V_{n}^{W}=\underset{i<n}{\cup }\left\{T_{n}-l*T_{i}\mathrm{for}\;\mathrm{all}\left\lceil \frac{T_{n}-WL_{n-1}}{T_{i}}\right\rceil \leq l\leq \left\lfloor \frac{T_{n}}{T_{i}}\right\rfloor \right\}.$$

By Equations (17) and (18), the worst-case slack time $S_{n}^{W}$ of $\tau_{n}$ can be obtained by

(19) $$S_{n}^{W}=\max_{t\in V_{n}^{W}}t-\sum_{i<n}\left\lceil \frac{t}{T_{i}}\right\rceil *C_{i}.$$

Based on Equation (18), the exact worst-case slack time of the lowest-priority task in a task set can be obtained in pseud-polynomial complexity with Equation (19), i.e., Lemma 3.

**Lemma 3.**  *Consider a schedulable task set $\Gamma =\{\tau_{1},\ldots ,\tau_{n}\}$ on a uniprocessor under the rate monotonic (RM) policy. Here, tasks in* Γ *are ordered in decreasing priority. We can obtain the exact worst-case slack time of task $\tau_{n}$ with the computational complexity $O(n*T_{n}/T_{1})$.*

**Theorem 7.**  *Consider a schedulable task set $\Gamma =\{\tau_{1},\tau_{2},\ldots ,\tau_{n}\}$ on a uniprocessor under the rate monotonic (RM) policy. Here, tasks in* Γ *are ordered in decreasing priority. Let $J_{n}^{B}$ be a job of task $\tau_{n}$ whose succeeding job is released concurrently with the jobs of all tasks with higher priorities, and the release time of job $J_{n}^{B}$ is denoted as $r_{n}^{B}$. Let $r_{n}^{B}-t_{\mathrm{end}}$ be the end time of the last available slack for job $J_{n}^{B}$ not later than $r_{n}^{B}$. Then, the amount of available slack time for $J_{n}^{B}$ can be expressed as follows:*

(20) $$S_{n}^{B}=T_{n}+t_{\mathrm{end}}-\sum_{i<n}\left\lfloor \frac{T_{n}+t_{\mathrm{end}}}{T_{i}}\right\rfloor *C_{i}.$$

**Figure 5:**
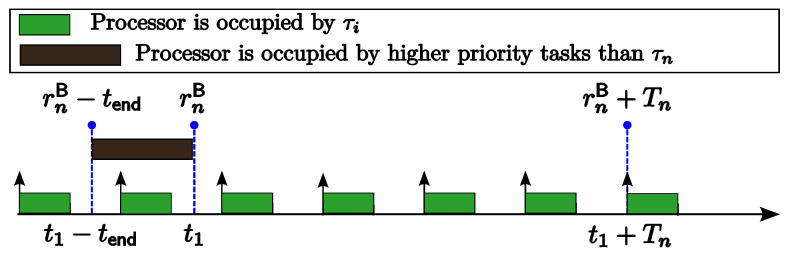
Workload of higher-priority tasks $\tau_{i}$ (i<n) during $\left[r_{n}^{B}-t_{\mathrm{end}},r_{n}^{B}+T_{n}\right)$.

**Proof.**  Suppose that $J_{n}^{B}$ is released at time $t_{1}$ and its succeeding job is released simultaneously with the jobs of all tasks with higher priorities at time $t_{1}+T_{n}$. According to Theorem 5, job $J_{n}^{B}$ will experience the best-case slack time for the task $\tau_{n}$. Figure 5 gives the workload of higher-priority tasks $\tau_{i}$ (i<n) during $\left[r_{n}^{B}-t_{\mathrm{end}},r_{n}^{B}+T_{n}\right)$. Since $r_{n}^{B}-t_{\mathrm{end}}$ is the end time of the last available slack for job $J_{n}^{B}$ not later than $r_{n}^{B}$, from Figure 5, we can observe that the jobs of all tasks with higher priorities released not later than $r_{n}^{B}-t_{\mathrm{end}}$ are finished before time $r_{n}^{B}-t_{\mathrm{end}}$ and there is no available slack for $J_{n}^{B}$ during the interval $\left[r_{n}^{B}-t_{\mathrm{end}},r_{n}^{B}\right)$. Moreover, because the succeeding job of $J_{n}^{B}$ is released concurrently with the jobs of all tasks with higher priorities, for each higher-priority task $\tau_{i}$ (i<n), the number of jobs that need to be executed during the interval $\left[r_{n}^{B}-t_{\mathrm{end}},r_{n}^{B}+T_{n}\right)$ is $\left\lfloor \left(T_{n}+t_{\mathrm{end}}\right)/T_{i}\right\rfloor$. Hence, the workload of the higher-priority tasks during $\left[r_{n}^{B},r_{n}^{B}+T_{n}\right)$ is $\sum_{i<n}\left\lfloor \left(T_{n}+t_{\mathrm{end}}\right)/T_{i}\right\rfloor *C_{i}-t_{\mathrm{end}}$. Therefore, the amount of available slack for $J_{n}^{B}$ can be expressed with Equation (20).   □

Similar to the worst-case slack time analysis of $\tau_{n}$, $t_{\mathrm{end}}$ for job $J_{n}^{B}$ can be expressed as

(21) $$t_{\mathrm{end}}=\underset{t\in V_{n}^{B}}{\arg\min}T_{n}+t-\sum_{i<n}\left\lfloor \frac{T_{n}+t}{T_{i}}\right\rfloor *C_{i}.$$

where $V_{n}^{B}$ is the search space for $t_{\mathrm{end}}$ of job $J_{n}^{B}$, and it can be expressed as

(22) $$V_{n}^{B}=\underset{i<n}{\cup }\left\{l*T_{i}-T_{n}\mathrm{for}\;\mathrm{all}\left\lceil \frac{T_{n}}{T_{i}}\right\rceil \leq l\leq \left\lfloor \frac{T_{n}+WL_{n-1}}{T_{i}}\right\rfloor \right\}.$$

From Equation (21), we can obtain the best-case slack time $S_{n}^{B}$ for task $\tau_{n}$ as

(23) $$S_{n}^{B}=\min_{t\in V_{n}^{B}}T_{n}+t-\sum_{i<n}\left\lfloor \frac{T_{n}+t}{T_{i}}\right\rfloor *C_{i}.$$

Based on Equation (22), the exact best-case slack time for the lowest-priority task in a task set can be obtained in pseudo-polynomial complexity with Equation (23), i.e., Lemma 4.

**Lemma 4.**  *Consider a schedulable task set $\Gamma =\{\tau_{1},\ldots ,\tau_{n}\}$ on a uniprocessor under the rate monotonic (RM) policy. Here, tasks in* Γ *are ordered in decreasing priority. We can obtain the exact best-case slack time of $\tau_{n}$ with the computational complexity $O(n*T_{n}/T_{1})$.*

By the definition of the slack variation-based harmonic index (i.e., Equation (14)), we can derive Theorem 8 by using Lemmas 3 and 4.

**Theorem 8.**  *Consider a schedulable task set $\Gamma =\{\tau_{1},\ldots ,\tau_{n}\}$ on a uniprocessor under rate monotonic (RM) policy. Here, tasks in* Γ *are ordered in decreasing priority. The slack variation-based harmonic index of* Γ *can be derived with the computational complexity $O(n*T_{n}/T_{1})$.*

## 4. Enhanced Harmonic Task Partitioning

With the slack variation-based harmonic index, we propose two efficient partitioned scheduling algorithms to improve the system utilization via strategically allocating the workload among processors by leveraging the task harmonic relationship. In the following, we first introduce an algorithm (named EHAP-SV) to optimize the system usage by identifying tasks with high harmonicity and grouping them into a single processor. However, the EHAP-SV algorithm suffers from the “fragmentation” problem as it does not consider some workload characteristics in the task allocation. To address this issue, we present the WAHP-SV algorithm, a workload-aware harmonic partitioning method. WAHP-SV incorporates both harmonicity and workload characteristics into the scheduling approach to address the “fragmentation” issue in the EHAP-SV algorithm.

### 4.1. Harmonic Partitioning Based on Slack Analysis

Before presenting our harmonic-aware partitioning method, let us discuss the limitations of the current harmonicity-aware partitioning approach using an example. In [14], a harmonicity-aware task partitioning algorithm (named HAPS) is proposed. HAPS measures the harmonicity degree of a task set by using the utilization change-based harmonic index and allocates harmonic tasks to an identical processor. It has been demonstrated that HAPS outperforms traditional bin-packing approaches. However, HAPS cannot optimize the schedulability of certain task sets, since the harmonic index used by HAPS is too tight to effectively quantify the harmonicity of some task sets (see Example 1). In the following, we will illustrate the effect of the inaccurate harmonicity quantification on the harmonicity-aware task partitioning with an example.

**Example 3.**  *Let us consider a task set $\Gamma =\{\tau_{1},\tau_{2},\tau_{3},\tau_{4},\tau_{5}\}$, given in Table 3, to be executed on processors $\Pi =\{\pi_{1},\pi_{2}\}$ under RM policy. When the HAPS strategy is employed to partition this task set, HAPS considers that tasks $\tau_{1}$, $\tau_{2}$, and $\tau_{3}$ are not harmonic, and it partitions all tasks into three groups, $\left\{\tau_{1},\tau_{4}\right\}$, $\left\{\tau_{3},\tau_{5}\right\}$, $\left\{\tau_{2}\right\}$, one by one. This implies that HAPS requires three processors to schedule* Γ*. Hence, HAPS fails to partition* Γ *for processors $\pi_{1}$ and $\pi_{2}$, even though a high overall capacity is still available on processors $\pi_{1}$ and $\pi_{2}$. This is primarily because the harmonic index used by HAPS is too tight to effectively quantify the harmonicity of task set $\left\{\tau_{1},\tau_{2},\tau_{3}\right\}$ from the perspective of the system utilization. In the following, we propose a feasible partitioning method for* Γ*. This strategy considers the harmonicity characteristic through slack analysis during task deployment, thereby enhancing system utilization (refer to Example 4).*

To enhance the processor utilization through efficient harmonic-aware partitioning based on effective harmonicity quantification, we propose an enhanced harmonic-aware partitioning algorithm using the slack variation-based harmonic index (named EHAP-SV). In EHAP-SV, some candidate subsets are generated first by identifying the harmonic tasks with the slack variation-based harmonic index. Then, the highest utilization subset is picked for assignment to a processor to enhance system utilization.

The EHAP-SV algorithm is given in Algorithm 1. It begins by initializing the required processor number *k* to zero, and then, proceeds to allocate tasks group by group by using the slack variation-based harmonic index (lines 3–42). Every task $\tau_{i}\in \Gamma$ is considered to be the host task in a sub-task set $\Gamma_{i}^{\mathrm{sub}}$ (line 5), and remaining tasks are picked to be added to $\Gamma_{i}^{\mathrm{sub}}$ until all feasible tasks have been exhausted (lines 6–30). To ensure the schedulability of the assigned task sets, a schedulability analysis can be performed using the efficient exact schedulability test approach proposed in [22]. During the generation of the sub-task set $\Gamma_{i}^{\mathrm{sub}}$, the feasible task with the minimum harmonic index is selected first (lines 14–17). For multiple feasible tasks with a minimum harmonic index, the task with higher utilization is selected first (lines 18–20). After all subsets are generated, the subsets with the highest utilization are selected and assigned to a core (lines 32–40). Then, these tasks are deleted from Γ (line 41). This process is iterated for the remaining tasks in task set Γ until all tasks have been allocated to processors.

**Theorem 9.**  *For a task set $\Gamma =\{\tau_{1},\ldots ,\tau_{n}\}$, if EHAP-SV partitions it onto m processors $\Pi =\{\pi_{1},\ldots ,\pi_{m}\}$ with success and schedules it under the RM scheduling policy, all tasks in* Γ *can satisfy their deadlines.*

**Proof.**  Let us take processor $\pi_{j}\in \Pi$ and its assigned task set $\Gamma (\pi_{j})$ into consideration. As indicated in line 12 in Algorithm 1, it is schedulable for $\Gamma (\pi_{j})$ to be executed on processor $\pi_{j}$ according to RM scheduling policy. This implies that the task set assigned to each processor is schedulable.    □

From Theorem 9, we can conclude that the task allocation produced by EHAP-SV consistently guarantees that all tasks in Γ meet their deadlines.
**Algorithm 1** EHAP-SV algorithm.**Input:** 
Task set $\Gamma =\{\tau_{1},\ldots ,\tau_{n}\}$, multiprocessor platform Π.**Output:** 
Task partitions $\left\{\Gamma \left(\pi_{1}\right),\ldots ,\Gamma \left(\pi_{k}\right)\right\}$.**Goal:** 
To partition task set Γ on multiprocessor platform Π.  1:k←0  2:**while** Γ≠∅ **do**  3:   k←k+1  4:   **for** each task $\tau_{i}\in \Gamma$ **do**  5:     $\Gamma_{i}^{\mathrm{sub}}\leftarrow \left\{\tau_{i}\right\}$  6:     $\Gamma_{\mathrm{untest}}\leftarrow \Gamma \setminus \left\{\tau_{i}\right\}$  7:     **while** $\Gamma_{\mathrm{untest}}\neq \emptyset$ **do**  8:        $\tau_{\mathrm{selected}}\leftarrow \mathrm{NIL}$  9:        $U_{\mathrm{selected}}\leftarrow 0$10:        $H^{\min}\leftarrow 1$11:        **for** each task $\tau_{j}\in \Gamma_{\mathrm{untest}}$ **do**12:          **if** It is feasible for $\Gamma_{i}^{\mathrm{sub}}\cup \left\{\tau_{j}\right\}$ **then**13:             $H_{j}\leftarrow H^{\mathrm{slack}}\left(\Gamma_{i}^{\mathrm{sub}}\cup \left\{\tau_{j}\right\}\right)$14:             **if** $H^{\min}>H_{j}$ **then**15:               $H^{\min}\leftarrow H_{j}$16:               $\tau_{\mathrm{selected}}\leftarrow \tau_{j}$17:               $U_{\mathrm{selected}}\leftarrow U_{j}$18:             **else if** $H^{\min}=H_{j}$ and $U_{\mathrm{selected}}<U_{j}$19:               $\tau_{\mathrm{selected}}\leftarrow \tau_{j}$ **then**20:               $U_{\mathrm{selected}}\leftarrow U_{j}$21:             **end if**22:          **else**23:             $\Gamma_{\mathrm{untest}}\leftarrow \Gamma_{\mathrm{untest}}\setminus \left\{\tau_{j}\right\}$24:          **end if**25:        **end for**26:        **if** $\tau_{\mathrm{selected}}\neq \mathrm{NIL}$ **then**27:          $\Gamma_{i}^{\mathrm{sub}}\leftarrow \Gamma_{i}^{\mathrm{sub}}\cup \left\{\tau_{\mathrm{selected}}\right\}$28:          $\Gamma_{\mathrm{untest}}\leftarrow \Gamma_{\mathrm{untest}}\setminus \left\{\tau_{\mathrm{selected}}\right\}$29:        **end if**30:     **end while**31:   **end for**32:   $\Gamma_{\mathrm{selected}}\leftarrow \emptyset$33:   $U^{\max}\leftarrow 0$34:   **for** each task $\tau_{i}\in \Gamma$ **do**35:     **if** $U^{\max}<U_{\Gamma_{i}^{\mathrm{sub}}}$ **then**36:        $\Gamma_{\mathrm{selected}}\leftarrow \Gamma_{i}^{\mathrm{sub}}$37:        $U^{\max}\leftarrow U_{\Gamma_{i}^{\mathrm{sub}}}$38:     **end if**39:   **end for**40:   $\Gamma \left(\pi_{k}\right)\leftarrow \Gamma_{\mathrm{selected}}$41:   $\Gamma \leftarrow \Gamma \setminus \Gamma_{\mathrm{selected}}$42:**end while**

From Algorithm 1, it is evident that the computational complexity of the EHAP-SV algorithm is primarily influenced by the feasibility test’s complexity, the complexity of computing the harmonic index based on slack variation, and the task set size. The feasibility tests can be achieved in pseudo-polynomial time with the WCRT analysis using the iterative technique [22]. In addition, from Theorem 8, we can obtain that the calculation of the slack variation-based harmonic index also exhibits pseudo-polynomial time complexity. Therefore, we can conclude that the EHAP-SV algorithm’s time complexity is pseudo-polynomial.

**Figure 6:**
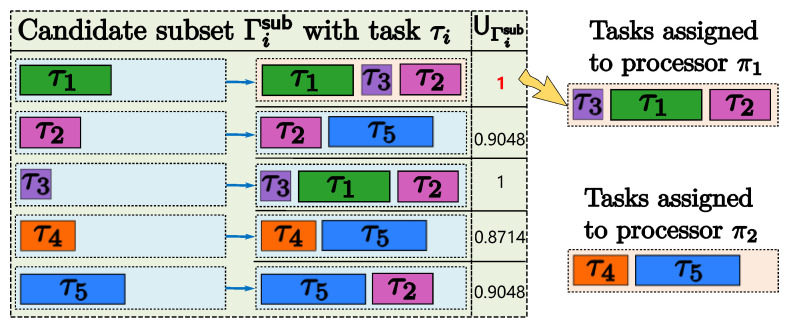
Task partitioning process with EHAP-SV.

**Example 4.**  *Let us consider the task set Γ given in Table 3 once again. As illustrated in Figure 6, it is the task partitioning process for* Γ *using EHAP-SV. We can see that EHAP-SV partitions all tasks in two groups, $\left\{\tau_{1},\tau_{2},\tau_{3}\right\}$ and $\left\{\tau_{4},\tau_{5}\right\}$, one by one. Consequently, EHAP-SV can allocate the task set* Γ *to two processors by effectively exploring the harmonic relationship based on its effective harmonicity quantification.*

### 4.2. Workload-Aware Harmonic Partitioning

The EHAP-SV method can optimize system usage by leveraging the harmonic relationship to assign harmonic tasks to the same processor. However, it may face performance degradation when dealing with heavy-utilization tasks, as it does not consider certain workload characteristics during the task allocation. To address this issue, we design a metric that combines harmonic relationship exploration with workload awareness to guide task allocation, and design a workload-aware harmonic task partitioning method (named WAHP-SV).

Algorithm 2 gives the WAHP-SV algorithm. Unlike EHAP-SV, we introduce a new metric, denoted as $\nu_{j}$, for each task $\tau_{j}$. This metric is employed for determining tasks that exhibit both high harmonicity and high workload for generating candidate subsets in WAHP-SV. For a sub-task set $\Gamma_{i}^{\mathrm{sub}}$ and a task $\tau_{j}$, the metric $\nu_{j}$ is defined by

(24) $$\nu_{j}=U_{j}-H^{\mathrm{slack}}\left(\Gamma_{i}^{\mathrm{sub}}\cup \left\{\tau_{j}\right\}\right).$$

where $U_{j}$ is the utilization of task $\tau_{j}$ and it is used for workload awareness. $H^{\mathrm{slack}}\left(\Gamma_{i}^{\mathrm{sub}}\cup \left\{\tau_{j}\right\}\right)$ is a measure of the harmonicity for task set $\Gamma_{i}^{\mathrm{sub}}\cup \left\{\tau_{j}\right\}$ and it is used for harmonic awareness. In the task assignment, the tasks with higher $\nu_{j}$ are prioritized (lines 13–15).

**Theorem 10.**  *For a task set* Γ*, if WAHP-SV successfully partitions it onto m processors $\Pi =\{\pi_{1},\ldots ,\pi_{m}\}$ and schedules it under the RM scheduling policy, all tasks in* Γ *can satisfy their deadlines.*

**Proof.**  Let us take processor $\pi_{j}\in \Pi$ and its assigned task set $\Gamma (\pi_{j})$ into consideration. As indicated in line 11 in Algorithm 2, it is feasible for $\Gamma (\pi_{j})$ to be executed on processor $\pi_{j}$ under the RM policy. This indicates that it is schedulable for the task set on each processor.    □

From Theorem 10, we can conclude that the task allocation produced by WAHP-SV consistently guarantees that all tasks in Γ meet their deadlines.

From Algorithm 2, it is evident that the time complexity of the WAHP-SV algorithm is identical to that of the EHAP-SV algorithm. Thus, the time complexity of the WAHP-SV algorithm is also pseudo-polynomial.

It is worth noting that WAHP-SV can also partition the task set Γ given in Table 3 for two processors. Furthermore, the experimental evaluation demonstrates that WAHP-SV outperforms EHAP-SV in terms of schedulability, attributed to its ability to be aware of workload and explore harmonic relationships.
**Algorithm 2** WAHP-SV algorithm.**Input:** 
Task set $\Gamma =\{\tau_{1},\ldots ,\tau_{n}\}$, multiprocessor platform Π.**Output:** 
Task partitions $\left\{\Gamma \left(\pi_{1}\right),\ldots ,\Gamma \left(\pi_{k}\right)\right\}$.**Goal:** 
To partition task set Γ on multiprocessor platform Π.  1:k←0  2:**while** Γ≠∅ **do**  3:   k←k+1  4:   **for** each task $\tau_{i}\in \Gamma$ **do**  5:     $\Gamma_{i}^{\mathrm{sub}}\leftarrow \left\{\tau_{i}\right\}$  6:     $\Gamma_{\mathrm{untest}}\leftarrow \Gamma \setminus \left\{\tau_{i}\right\}$  7:     **while** $\Gamma_{\mathrm{untest}}\neq \emptyset$ **do**  8:        $\tau_{\mathrm{selected}}\leftarrow \mathrm{NIL}$  9:        $\nu^{\max}\leftarrow 0$10:        **for** each task $\tau_{j}\in \Gamma_{\mathrm{untest}}$ **do**11:          **if** It is feasible for $\Gamma_{i}^{\mathrm{sub}}\cup \left\{\tau_{j}\right\}$ **then**12:             $\nu_{j}\leftarrow U_{j}-H^{\mathrm{slack}}\left(\Gamma_{i}^{\mathrm{sub}}\cup \left\{\tau_{j}\right\}\right)$13:             **if** $\nu^{\max}<\nu_{j}$ **then**14:               $\tau_{\mathrm{selected}}\leftarrow \tau_{j}$15:               $\nu^{\max}\leftarrow \nu_{j}$16:             **end if**17:          **else**18:             $\Gamma_{\mathrm{untest}}\leftarrow \Gamma_{\mathrm{untest}}\setminus \left\{\tau_{j}\right\}$19:          **end if**20:        **end for**21:        **if** $\tau_{\mathrm{selected}}\neq \mathrm{NIL}$ **then**22:          $\Gamma_{i}^{\mathrm{sub}}\leftarrow \Gamma_{i}^{\mathrm{sub}}\cup \left\{\tau_{\mathrm{selected}}\right\}$23:          $\Gamma_{\mathrm{untest}}\leftarrow \Gamma_{\mathrm{untest}}\setminus \left\{\tau_{\mathrm{selected}}\right\}$24:        **end if**25:     **end while**26:   **end for**27:   $\Gamma_{\mathrm{selected}}\leftarrow \emptyset$28:   $U^{\max}\leftarrow 0$29:   **for** each task $\tau_{i}\in \Gamma$ **do**30:     **if** $U^{\max}<U_{\Gamma_{i}^{\mathrm{sub}}}$ **then**31:        $\Gamma_{\mathrm{selected}}\leftarrow \Gamma_{i}^{\mathrm{sub}}$32:        $U^{\max}\leftarrow U_{\Gamma_{i}^{\mathrm{sub}}}$33:     **end if**34:   **end for**35:   $\Gamma \left(\pi_{k}\right)\leftarrow \Gamma_{\mathrm{selected}}$36:   $\Gamma \leftarrow \Gamma \setminus \Gamma_{\mathrm{selected}}$37:**end while**

## 5. Experimental Evaluation

To assess the effectiveness of our enhanced harmonic task partitioning methods, we implemented eight algorithms in the experiment, which are listed below:

- BFDU: an extension of the best-fit (BF) strategy, where tasks are ordered in decreasing utilization;
- WFDU: an extension of the worst-fit (WF) strategy, where tasks are ordered in decreasing utilization;
- FFDU: an extension of the first-fit (FF) strategy, where tasks are ordered in decreasing utilization;
- PSER: a harmonic partition scheduling algorithm based on the period scaling from [14];
- HAPS: a harmonic partition scheduling algorithm with the harmonic index based on utilization change from [14];
- ENSE: an ensemble method of BFDU, WFDU, FFDU, PSER, and HAPS;
- EHAP-SV: our enhanced harmonic partition scheduling algorithm with harmonic index based on slack variation;
- WAHP-SV: our workload-aware harmonic partition scheduling algorithm with harmonic index based on slack variation.

**Objectives**. Our evaluation has two primary goals: to compare the performance of our enhanced harmonic task partitioning algorithms with existing algorithms regarding schedulability; and to evaluate the required number of processors for different partition approaches.

### 5.1. Workload

To evaluate the performance of various methods in relation to system utilization, we synthesized two types of task sets (i.e., light task sets and general task sets) by the UUniFast approach from [23]. For the light task sets, task utilization is uniformly distributed within [0, 0.5], and for general task sets, task utilization is evenly distributed within [0, 1]. The task period is evenly distributed within [100, 1000]. For a task set Γ, we define its normalized utilization $U_{\mathrm{nor}}\left(\Gamma \right)$ as

(25) $$U_{\mathrm{nor}}\left(\Gamma \right)=\frac{U_{\Gamma }}{m}.$$

where *m* is the processor number and $U_{\Gamma }$ is the utilization of Γ. We consider three values for *m*: 4, 8, and 16.

With the above parameters, we created task sets with normalized utilization $U_{\mathrm{nor}}$ ranging from 0.7 to 0.975 with an increment of 0.025. We synthesized 1000 task sets for each $U_{\mathrm{nor}}$.

### 5.2. Results

**Figure 7:**
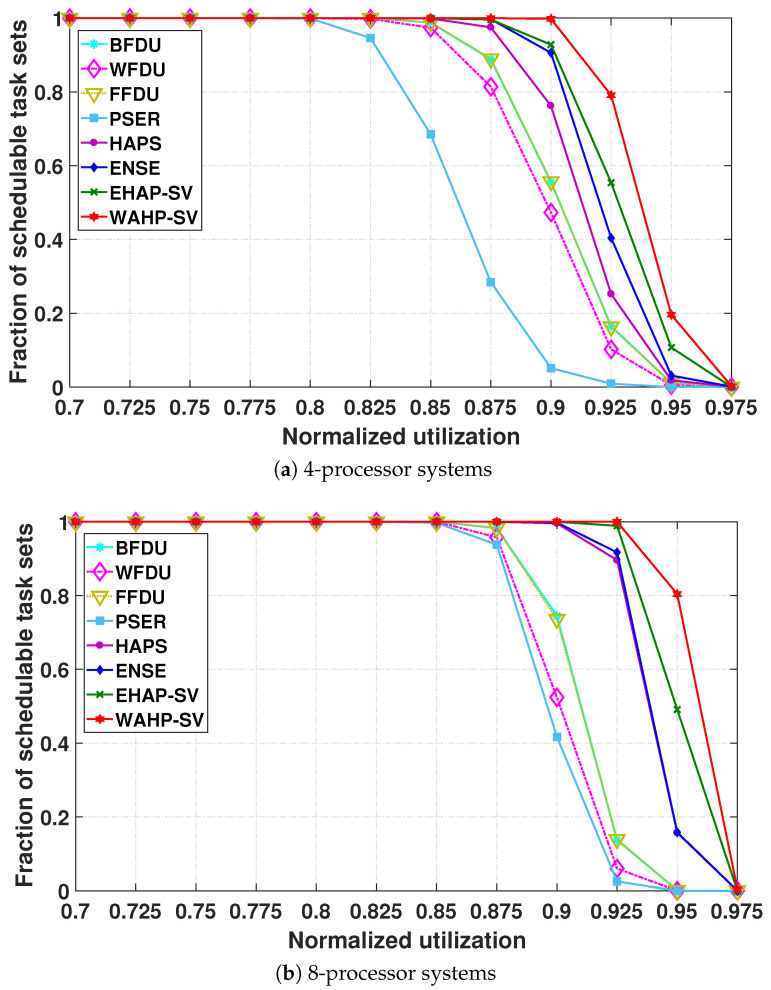

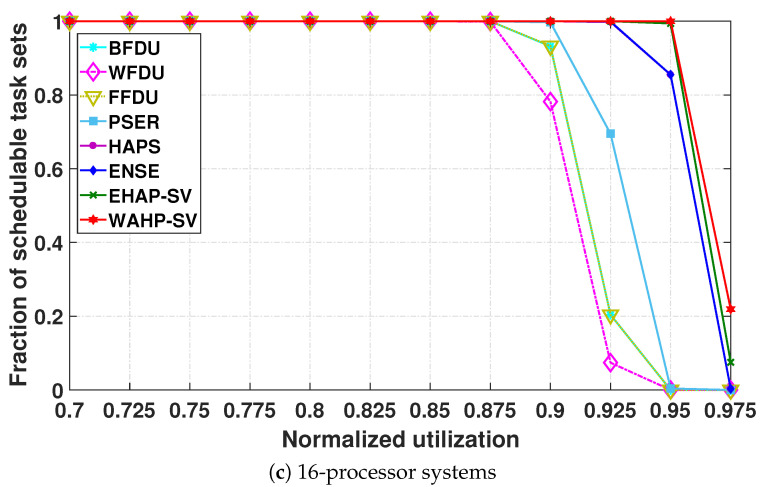
Fraction of schedulable tasks sets for light task sets.

**Figure 8:**
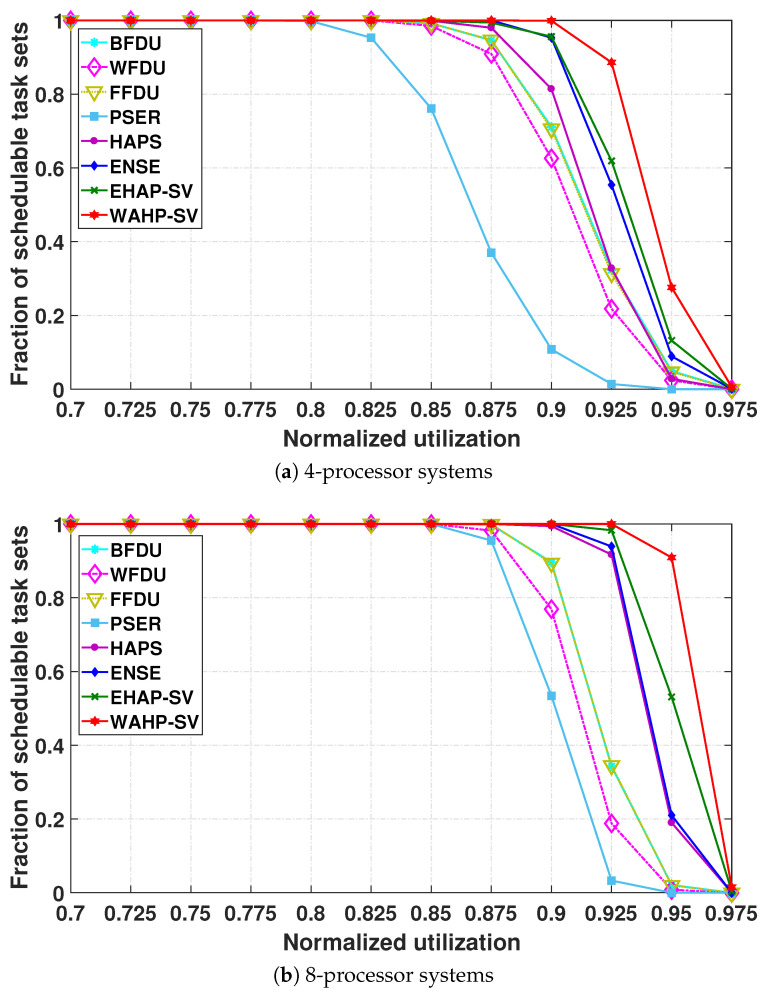

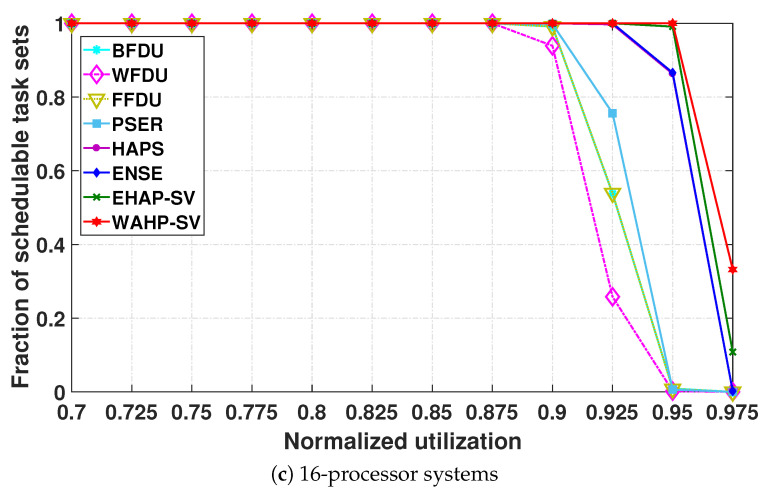
Fraction of schedulable tasks sets for general task sets.

**Schedulability.** Figure 7 and Figure 8 illustrate the schedulable task set fraction plotted against the normalized system utilization in systems with 4 processors, 8 processors, and 16 processors for light task sets and general task sets, respectively.

We make several observations regarding the performance of the existing algorithms. First, HAPS consistently outperforms BFDU, WFDU, FFDU, and PSER in most cases. This is because HAPS can take advantage of harmonics to enhance the schedulability of the system by taking into account both the period and utilization of each task in the quantification of harmonicity, while BFDU, WFDU, and FFDU overlook the effect of harmonic relationship exploration on the system schedulability and PSER only considers the relationship among periods of tasks in the harmonicity quantification. Second, BFDU, WFDU, and FFDU outperform PSER in the systems with four and eight processors for all types of task sets. This is because BFDU, WFDU, and FFDU can enhance the system schedulability by leveraging the task workload characteristic, especially for the general task sets with some heavy tasks. Third, the schedulability enhancement of BFDU, WFDU, and FFDU over PSER is reduced as the processor number rises, and they perform worse than PSER for 16-processor systems. This can be attributed to the fact that with more processors there are more tasks in a task set, providing more chances for PSER to enhance system schedulability by exploiting the task period relationships.

Our primary result is that EHAP-SV and WAHP-SV consistently outperform all existing partitioned scheduling methods owing to the effective harmonicity quantification of the slack variation-based harmonic index. Note that EHAP-SV and WAHP-SV also always outperform the ensemble method (i.e., ENSE) of BFDU, WFDU, FFDU, PSER, and HAPS. This demonstrates that EHAP-SV and WAHP-SV can find additional schedulable task sets that have not been found so far with existing methods. The performance enhancement grows with increasing processor number, depicting the scalability of EHAP-SV and WAHP-SV. For 16-processor systems, when the system utilization is 0.975, WAHP-SV schedules about 21.9% and 33.2% of task sets for light task sets and general task sets, respectively, while all existing methods schedule almost none of them. Even without workload awareness, for general task sets, when the system utilization is 0.95, the performance improvement of EHAP-SV over the ensemble method ENSE due to the effective harmonic quantification is as much as 48.3% in a four-processor system and 152.9% in an eight-processor system. In addition, we can also see that WAHP-SV always outperforms EHAP-SV in all test cases, and the schedulability improvement of WAHP-SV over EHAP-SV becomes more significant as the task set utilization increases, especially for general task sets. As system utilization increases, tasks with higher utilization are prone to be part of a general task set, and WAHP-SV can significantly improve the partitioning of such task sets through workload awareness. In particular, when system utilization is 0.95 in four-processor systems, the schedulable ratio achieved by WAHP-SV is nearly double that of EHAP-SV.

**Figure 9:**
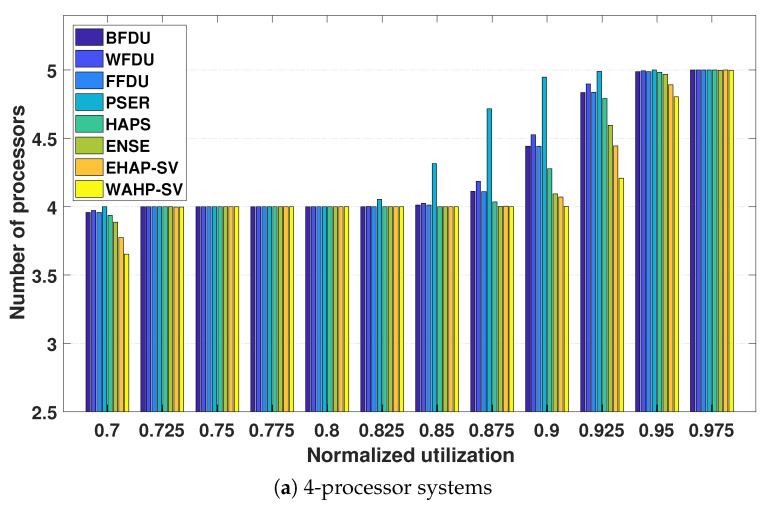

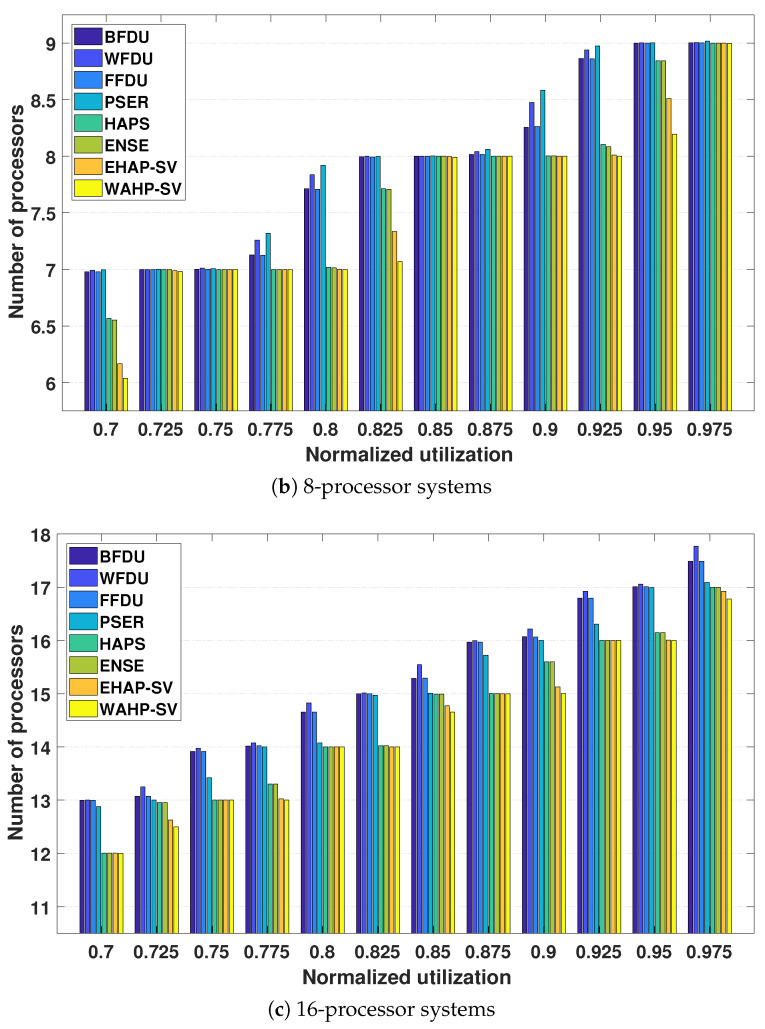
Processor requirements for light task sets.

**Figure 10:**
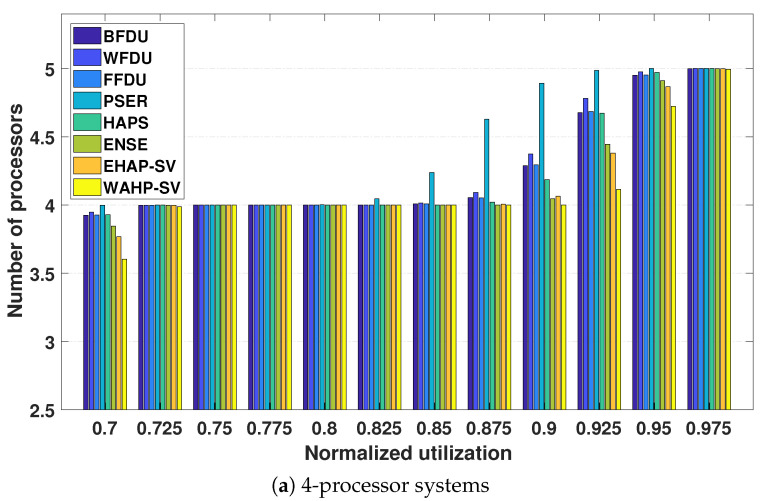

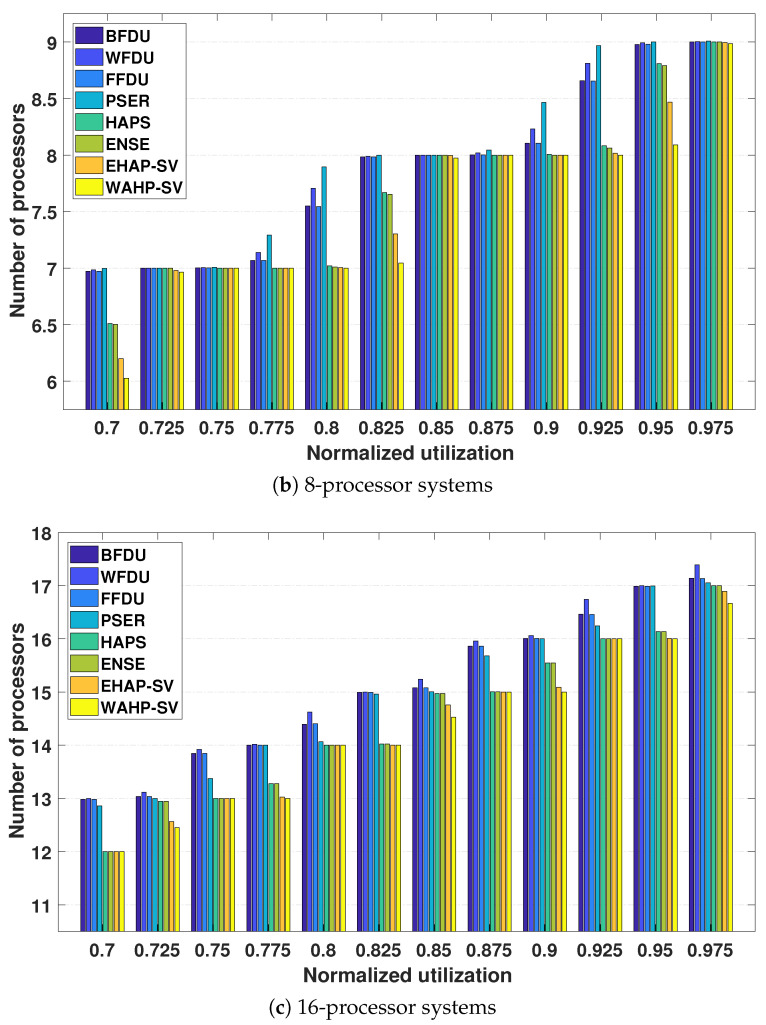
Processor requirements for general task sets.

**Number of processors.** Figure 9 and Figure 10 illustrate the relationship between the required processor number and normalized system utilization for systems with 4, 8, and 16 processors for light task sets and general task sets, respectively.

From the experimental results, we can observe that both EHAP-SV and WAHP-SV outperform all existing methods including the ensemble method ENSE in most test cases. This can be attributed to the fact that EHAP-SV and WAHP-SV enhance the performance through efficient exploration of harmonic relationships based on effective quantification of harmonicity. The results also demonstrate that WAHP-SV outperforms EHAP-SV in some test cases, owing to its effective integration of harmonic relationship exploration and workload awareness. Additionally, we can observe that WAHP-SV can save up to one processor compared to the non-harmonic partitioned scheduling approaches (i.e., BFDU, WFDU, and FFDU) in 8-processor system and 16-processor systems for both light and general task sets.

## 6. Conclusions and Future Work

As one of the primary approaches in multiprocessor scheduling, partitioned scheduling has been widely accepted in both academic and industrial fields of IoT to efficiently process substantial streams of sensor-generated data while maintaining real-time performance. In this study, we propose an enhanced harmonic partitioned scheduling method that is based on efficient harmonic relationship exploration. In our approach, we designed a harmonic index to guide the task partition by quantifying the task set harmonicity in terms of the maximum variation in the slack time of the lowest-priority task in the task set. With our proposed harmonic index, we presented a harmonic task partitioning scheme (named HWAP-SV) for periodic real-time tasks scheduled on multiprocessors following RM policy. Furthermore, to alleviate the problem of “fragmentation” in the pure harmonic task partitioning method, we also proposed a workload-aware harmonic task partitioning scheme called WAHP-SV by designing a metric that combines harmonic relationship exploration with workload awareness to strategically allocate workloads among processors. Our evaluation shows that both EHAP-SV and WHAP-SV consistently surpass the state-of-the-art partitioned scheduling methods. We believe that similar results can be obtained for the mixed-criticality task model and directed acyclic graph (DAG) task model owing to the generality of the slack variation-based harmonic index, and we would like to verify this in the future.

## Figures and Tables

**Table 1:** Major notation used in the paper.

Notation	Description
$C_{i}$	Worst-case execution time of task $\tau_{i}$
$T_{i}$	Period of task $\tau_{i}$
$D_{i}$	Relative deadline of task $\tau_{i}$
$J_{i,k}$	A job of task $\tau_{i}$
$r_{i,k}$	Release time of job $J_{i,k}$
$R_{i}$	Worst-case response time of task $\tau_{i}$
$U_{i}$, $U_{\Gamma }$	Utilization of task $\tau_{i}$ and task set Γ, respectively
$H_{\Gamma }$	Hyperperiod of task set Γ
$S_{i,k}$	Slack time for a job $J_{i,k}$ of task $\tau_{i}$
$S_{i}^{W}$	Worst-case slack time of task $\tau_{i}$
$S_{i}^{B}$	Best-case slack time of task $\tau_{i}$
$H^{\mathrm{uc}}\left(\Gamma \right)$	Utilization change-based harmonic index of task set Γ
$H^{\mathrm{slack}}\left(\Gamma \right)$	Slack variation-based harmonic index of task set Γ

**Table 2:** An example task set for harmonic index $H^{\mathrm{uc}}$.

Task	$C_{i}$	$T_{i}$	$T_{i}^{\prime }$
$\tau_{1}$	1	2	1.5
$\tau_{2}$	1	3	3
$\tau_{3}$	1	6	6

**Table 3:** Task parameters of an example task set.

Task	$C_{i}$	$T_{i}$	$D_{i}$	$U_{i}$
$\tau_{1}$	1	2	2	0.5
$\tau_{2}$	1	3	3	0.3333
$\tau_{3}$	1	6	6	0.1667
$\tau_{4}$	1.5	5	5	0.3
$\tau_{5}$	4	7	7	0.5714

## Data Availability

Data are contained within the article.
